# Mathematical ****Model of Glucagon Kinetics for the Assessment of Insulin-Mediated Glucagon Inhibition During an Oral Glucose Tolerance Test

**DOI:** 10.3389/fendo.2021.611147

**Published:** 2021-03-22

**Authors:** Micaela Morettini, Laura Burattini, Christian Göbl, Giovanni Pacini, Bo Ahrén, Andrea Tura

**Affiliations:** ^1^ Department of Information Engineering, Università Politecnica delle Marche, Ancona, Italy; ^2^ Division of Obstetrics and Feto-Maternal Medicine, Department of Obstetrics and Gynecology, Medical University of Vienna, Vienna, Austria; ^3^ Metabolic Unit, CNR Institute of Neuroscience, Padova, Italy; ^4^ Department of Clinical Sciences, Faculty of Medicine, Lund University, Lund, Sweden

**Keywords:** alpha-cell insulin sensitivity, glucagon secretion, glucose challenge, minimal model, parameter estimation, glucose homeostasis

## Abstract

Glucagon is secreted from the pancreatic alpha cells and plays an important role in the maintenance of glucose homeostasis, by interacting with insulin. The plasma glucose levels determine whether glucagon secretion or insulin secretion is activated or inhibited. Despite its relevance, some aspects of glucagon secretion and kinetics remain unclear. To gain insight into this, we aimed to develop a mathematical model of the glucagon kinetics during an oral glucose tolerance test, which is sufficiently simple to be used in the clinical practice. The proposed model included two first-order differential equations -one describing glucagon and the other describing C-peptide in a compartment remote from plasma - and yielded a parameter of possible clinical relevance (i.e., S_GLUCA_(t), glucagon-inhibition sensitivity to glucose-induced insulin secretion). Model was validated on mean glucagon data derived from the scientific literature, yielding values for S_GLUCA_(t) ranging from -15.03 to 2.75 (ng of glucagon·nmol of C-peptide^-1^). A further validation on a total of 100 virtual subjects provided reliable results (mean residuals between -1.5 and 1.5 ng·L^-1^) and a negative significant linear correlation (r = -0.74, p < 0.0001, 95% CI: -0.82 – -0.64) between S_GLUCA_(t) and the ratio between the areas under the curve of suprabasal remote C-peptide and glucagon. Model reliability was also proven by the ability to capture different patterns in glucagon kinetics. In conclusion, the proposed model reliably reproduces glucagon kinetics and is characterized by sufficient simplicity to be possibly used in the clinical practice, for the estimation in the single individual of some glucagon-related parameters.

## Introduction

Glucagon is secreted from the pancreatic alpha cells and plays an important role in the maintenance of glucose homeostasis. In fact, glucagon and insulin interact to maintain euglycemia. The plasma glucose levels determine whether glucagon secretion or insulin secretion is activated or inhibited. Low plasma glucose and related decrease in plasma insulin stimulates glucagon secretion, which in turn promotes hepatic glucose production, through gluconeogenesis and glycogenolysis, to normalize the glucose levels (1–3). As reviewed in (4), regulation of glucagon secretion is a complex phenomenon and involves endocrine/paracrine mechanisms—the so-called “intra-islet interaction” (5, 6)—as well as intrinsic mechanisms in the alpha cell related to glucose sensing.

Glucagon also plays a role in the pathophysiology of type 2 diabetes (T2DM). Indeed, in patients with T2DM elevated plasma glucagon levels have been observed in the fasting state, and defective suppression of glucagon secretion exists in the postprandial state, resulting in elevated plasma glucagon levels (7), which have been shown to reflect an altered insulin inhibition of alpha-cell glucagon exocytosis (8). Such kind of alterations appears already at an early stage of T2DM development. In fact, defective suppression has been also found in impaired glucose tolerance (9). Moreover, increased fasting glucagon and delayed glucagon suppression have been shown to go along with insulin resistance in individuals with normal and impaired glucose regulation (10). The interest for the study of glucagon is also due to the reason that in patients with diabetes suffering for severe hypoglycemic events the administration of glucagon, by either injection or nasal intake, is an important therapeutic option (11). However, despite the relevance of glucagon in glucose metabolism and as pharmacological agent in glucometabolic diseases, some aspects of its secretion and kinetics remain unclear. To gain insight into this, we aimed to develop a mathematical model, with features adequate for possible use in the clinical settings.

A relatively large set of mathematical models were developed with focus on glucagon secretion at cellular level (12–21). Other models were developed for whole-body analyses, but they were complex and with the inclusion of several parameters hard to assess in the single individual, thus useful for simulation purposes rather than for clinical applications (22, 23). Similar considerations hold for studies where a glucagon model was included as a block of a more general model of blood glucose regulation, such as in studies (24, 25).

Studies presenting models analyzing glucagon kinetics for possible clinical applications are rare. One study analyzed the kinetics of glucagon administered exogenously (26), without however accounting for the interplay with insulin or glucose; another study performed similar analyses for the case of therapy based on glucagon (plus insulin) infusion (27). Some other studies developed models for the analysis of the glucagon challenge test, which is however not widely used (28, 29). The study (30) had purposes more similar to those of our study, but the developed model analyzed glucagon kinetics during an intravenous glucose tolerance test (IVGTT), or an insulin-infusion test. To our knowledge, no study has been focused on modeling the glucagon kinetics during an oral glucose tolerance test (OGTT), despite the fact that OGTT has remarkable advantages compared to the IVGTT (or other glucose tolerance tests) in terms of simplicity, and hence applicability in the clinical context.

The specific aim of this study was therefore to develop a mathematical model of the glucagon kinetics during an OGTT, which is sufficiently simple to be used in the clinical practice. For the model development, we exploited glucagon data derived from the study (31). In more details, our main aim was to develop a “minimal model” allowing estimation of glucagon-related parameters in single individuals, with specific interest for one parameter with considerable potential for clinical applications, i.e., the sensitivity to glucose-induced insulin secretion of the glucagon inhibition. This may be denoted as alpha-cell insulin sensitivity.

## Materials and Methods

### Model Formulation

#### Model Equations

The proposed mathematical model of glucagon kinetics during an OGTT is based on the hypothesis of the “intra-islet interaction” (5, 6). This hypothesis assumes that inhibition of glucagon secretion during an OGTT—which reflects at plasma level in a suppression of plasma glucagon concentration—is mainly determined by glucose-induced insulin secretion. To model insulin secretion, plasma C-peptide concentration has been exploited, since plasma C-peptide is the best marker of insulin secretion at plasma level. In fact, C-peptide is co-secreted with insulin by the beta cells but, differently from insulin, it is not significantly affected by degradation operated by the liver.

**Figure 1:**
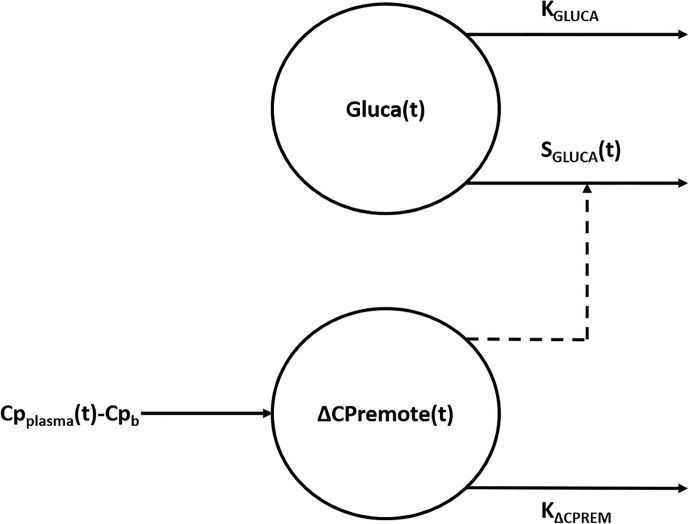
Compartmental representation of the model. The model is composed by two compartments, namely plasma glucagon compartment and remote (from plasma) C-peptide compartment. Suprabasal C-peptide concentration in the remote compartment [ΔCP_remote_(t)], used to model insulin secretion, exerts a control action on the concentration of glucagon in the plasma compartment [Gluca(t)] through the time-varying parameter S_GLUCA_(t). K_GLUCA_ represents the glucagon elimination rate from plasma whereas K_ΔCPREM_ is the C-peptide elimination rate from the remote compartment. ΔCP_remote_(t) represents a delayed version of suprabasal plasma C-peptide concentration measured during the test (CP_plasma_(t) - Cp_b_).

The model (**Figure 1**) is composed by two compartments, namely plasma glucagon compartment and remote (from plasma) C-peptide compartment, described by the following two ordinary differential equations:

(1) $$\frac{dGluca(t)}{dt}=-K_{GLUCA}\cdot Gluca(t)-S_{GLUCA}(t)\cdot \frac{d\Delta CP_{remote}(t)}{dt}Gluca(0)=Gluca_{b}$$

(2) $$\begin{array}{c} \frac{d\Delta CP_{remote}(t)}{dt}=-K_{\Delta CPREM}\cdot \Delta CP_{remote}(t)+(CP_{plasma}(t)-Cp_{b})\Delta CP_{remote}(0)=0 \end{array}$$

where Gluca(t) (ng·L^-1^) is the glucagon concentration in the plasma compartment, S_GLUCA_(t) (ng of glucagon·nmol of C-peptide^-1^) is a time-varying parameter expressing glucagon-inhibition sensitivity to glucose-induced insulin secretion during the test (i.e., alpha-cell insulin sensitivity) and K_GLUCA_ (min^-1^) represents the glucagon elimination rate from plasma due to clearance operated by liver and kidneys (32, 33); Gluca_b_ represents the basal plasma glucagon concentration measured during the test. ΔCP_remote_(t) (nmol·L^-1^) is the suprabasal C-peptide concentration in a compartment remote from plasma, which represents a delayed version of suprabasal plasma C-peptide concentration measured during the test, CP_plasma_(t) (nmol·L^-1^), with Cp_b_ being its basal value; K_ΔCPREM_ (min^-1^) is the C-peptide elimination rate from the remote compartment. The parameters to be estimated in the model in the single individual are: K_GLUCA_, S_GLUCA_(t), K_ΔCPREM_.

#### Structural Identifiability Analysis

Structural (*a priori*) identifiability of the model was tested by using DAISY (Differential Algebra for Identifiability of SYstems), a software tool that performs structural identifiability analysis for linear and nonlinear dynamic models described by polynomial or rational ordinary differential equations with either known or unknown initial conditions (34).

### Model Implementation

All the steps for model implementation are outlined in **Supplementary Figure 1**. Model has been implemented in MATLAB^®^ R2017b as a discrete-time system (considering *n* time points, equally spaced) and its response in terms of glucagon and remote C-peptide concentrations has been obtained using the *ltitr* built-in function. Model parameter vector p = [K_GLUCA_, S_GLUCA_(t), K_ΔCPREM_] has been estimated by solving, through the *lsqnonlin* function, the following nonlinear least-squares curve fitting problem:

(3) $$\underset{p}{min}\|f(p)\|{\|}_{2}^{2}=\underset{p}{min}[RSS+K_{GLUCA}+w_{1}\cdot S_{GLUCA}(t)+w_{2}\cdot (S_{GLUCA}(t)<0)]$$

where the first term represents the residual sum of squares (being the residuals the differences between model glucagon response and glucagon curve measured during the OGTT), whereas all the others are regularization terms added as constraints to provide more information to the problem and facilitate practical (*a posteriori*) identifiability. In particular, the second term has been added on the consideration that, during the OGTT, the main contribution to glucagon suppression is given by C-peptide action and not by glucagon clearance, thus K_GLUCA_ has to be small; the third and the last term, weighted through *w_1_* and *w_2_*, have been added to limit S_GLUCA_(t) rapid changes during the test and the number of samples where S_GLUCA_(t) becomes negative, respectively.

The optimal values of *w_1_* and *w_2_* were selected by an iterative procedure in which 100 possible combinations of values - considering 10 different values for *w_1_* and 10 different values for *w_2_*, randomly generated - were tested. A combination of values for *w_1_* and *w_2_* was considered acceptable if it provided mean residual values lower that 10%, otherwise was discarded. Such threshold was chosen on the consideration of the uncertainty on the glucagon measurements [10% in fact is a suitable value for the inter- and intra-assay coefficient of variation for glucagon (35)]. The optimal combination, among all the combinations tested, was the one that provided the lowest mean residual.

To find the global minimum among several possible local minima, a total of 10 runs of the *lsqnonlin* local solver from different starting point (randomly generated, between 0 and 1) have been performed using the *MultiStart* algorithm (36), which repeatedly runs the solver of the model starting from different initial values of the parameters, to improve the possibility of reaching the optimal solution.

The *trust-region-reflective* algorithm has been used by *lsqnonlin* to solve the problem and the following lower and upper bounds have been applied to the parameters: (0;1) for K_GLUCA_ and K_ΔCPREM_; (-∞; +∞) for S_GLUCA_(t). Function and step-size tolerances have been set to 10^-6^.

K_GLUCA_ and K_ΔCPREM_ estimates have been obtained considering the two parameters as constant for the whole test duration, whereas estimates have been obtained for S_GLUCA_(t) corresponding to the time samples where plasma glucagon and C-peptide concentrations have been measured. For all model parameters, the 95% CIs for the parameter estimates have been computed by using the *nlparci* function.

### Model Validation

#### Reported Mean Experimental Data

Mean experimental data reported by Pepino et al. (31) have been used to initially validate the model. The original study by Pepino et al. (31), from which the mean data have been drawn, included a total of seventeen non-diabetic subjects undergoing a 5-h 75-g OGTT. Plasma C-peptide and glucagon concentrations at 2 min before (considered as 0 min) and at 10, 20, 30, 60, 90, 120, 150, 180, 240, and 300 min after glucose ingestion have been considered. As indicated by Pepino et al. (31), plasma glucagon was measured by a direct, double-antibody radioimmunoassay (Millipore).

#### Virtual Population Generation

Starting from mean and standard deviation (SD) values reported by Pepino et al. (31), a total of 100 virtual subjects have been generated using sort of Monte Carlo approach (37). Each virtual subject is characterized by glucagon and C-peptide curves in response to an OGTT, in which glucagon and C-peptide concentrations at each time sample were randomly generated, based on normal distributions with mean and SD values derived by the study of Pepino et al. (31) (considering all samples within the 95% confidence interval, CI). Furthermore, in order to obtain curves that are physiologically plausible, some additional constraints have been added [e.g., sign of the derivative between two time samples equal to that of the reference curves (31)].

#### Ability of the Model to Capture Different Patterns in Glucagon Kinetics

The ability of the model to capture different patterns in glucagon kinetics was tested on four characteristics mean glucagon curves in response to a five-point 75g - OGTT reported by Gar et al. (38), as representative of the four clusters identified in glucagon curve shapes of individuals with different metabolic phenotypes (i.e., normal glucose tolerance, prediabetes, type 2 diabetes). The four clusters are characterized as follow: 1) Cluster 1 had high mean fasting glucagon and delayed suppression; 2) Cluster 2 had high mean fasting glucagon and rapid suppression; 3) Cluster 3 had low mean fasting glucagon and rapid suppression; 4) Cluster 4 had low mean fasting glucagon and a rising curve after glucose ingestion. For each cluster, mean glucagon and C-peptide concentrations measured at 0 min and at 30, 60, 90, 120 after glucose ingestion have been considered. As indicated by Gar et al. (38), plasma glucagon was measured with an ELISA (Glucagon ELISA; Mercodia, Uppsala, Sweden; catalog no: 10-1271-01).

#### Sensitivity to OGTT Duration

In order to test sensitivity of the S_GLUCA_(t) estimation to test duration, in the 100 virtual subjects, the S_GLUCA_(t) mediated on the full 5-h OGTT has been compared to S_GLUCA_(t) mediated considering shorter OGTTs (limiting the 5-h OGTT to 2-h and 3-h).

### Calculations and Statistical Analysis

The Kolmogorov‐Smirnov test was used to evaluate the hypothesis that each variable had a normal distribution with unspecified mean and variance. Values were reported as mean ± SD.

Over the 100 virtual subjects, linear regression analysis has been performed between mean S_GLUCA_(t) during the OGTT and the ratio between the suprabasal area under the curve of remote C-peptide (AUC_ΔCPremote_) to the area of glucagon below the basal condition (AUC_Gluca_); also, Pearson correlation coefficient (r) has been reported. In the case of skewed distributions tests were applied to the log-transformed values.

As regards the estimation of S_GLUCA_(t) according to the different OGTT durations, comparisons have been performed by means of a paired Student t-test in case of normally distributed variables or Wilcoxon signed-rank test in case of skewed distributed variables. The two-sided significance level was set at 5% (p < 0.05).

## Results

**Figure 2:**
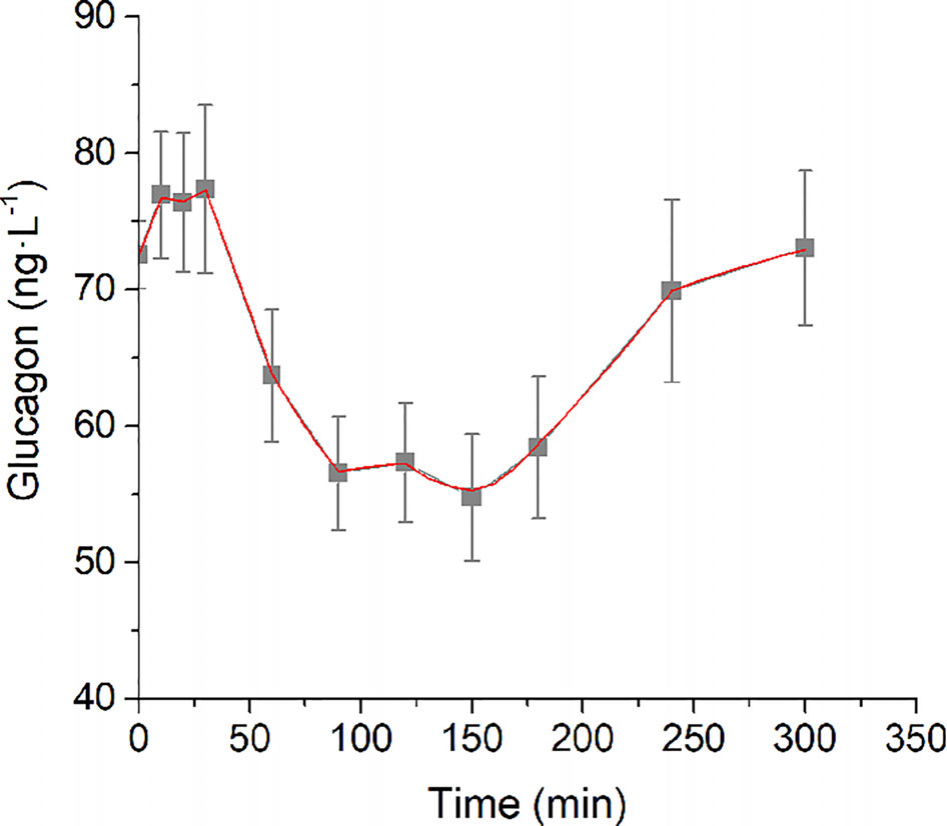
Best-fit results for model validation on reference mean experimental data by Pepino et al. (31). Grey squares are the reference experimental values (mean ± SD); red line is the model prediction.

**Figure 3:**
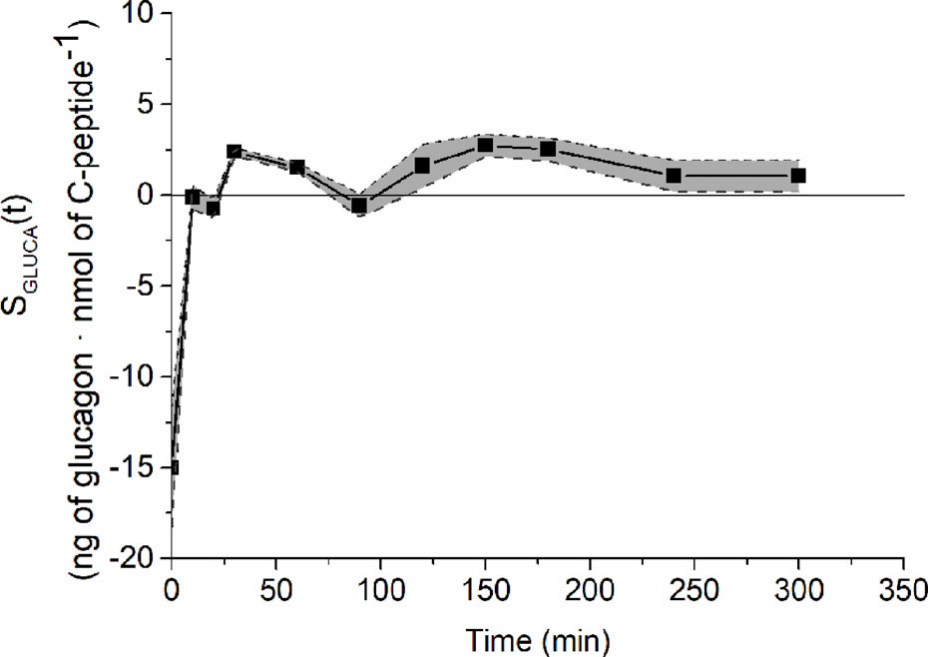
S_GLUCA_(t) temporal estimates on reference mean experimental data by Pepino et al. (31) (closed squares) and related CIs (dashed lines).

Analysis of structural (*a priori*) identifiability provided that the model was *a priori* identifiable (locally). Model validation on mean experimental data reported by Pepino et al. (31) provided the best fit shown in **Figure 2** and the parameter estimates (with related CIs) reported in **Table 1**. Trend of S_GLUCA_(t) during the whole OGTT is reported in **Figure 3**.

**Table 1:** Values and 95% CIs for the estimates of K_GLUCA_ (min^-1^), K_ΔCPREM_ (min^-1^), S_GLUCA_(t) (ng of glucagon·nmol of C-peptide^-1^) on reference mean experimental data by Pepino et al. (31).

Estimated parameter	Value	95% CI
**K_GLUCA_**	0.005	-0.002–0.013
**K_ΔCPREM_**	0.155	0.119–0.191
**S_GLUCA1_**	-15.03	-18.25 – -11.80
**S_GLUCA2_**	-0.11	-0.78–0.66
**S_GLUCA3_**	-0.73	-1.23 – -0.22
**S_GLUCA4_**	2.34	2.17–2.60
**S_GLUCA5_**	1.53	1.30–1.75
**S_GLUCA6_**	-0.58	-1.24–0.07
**S_GLUCA7_**	1.62	0.42–2.81
**S_GLUCA8_**	2.75	2.13–3.37
**S_GLUCA9_**	2.51	1.88–3.14
**S_GLUCA10_**	1.07	0.22–1.93

**Figure 4:**
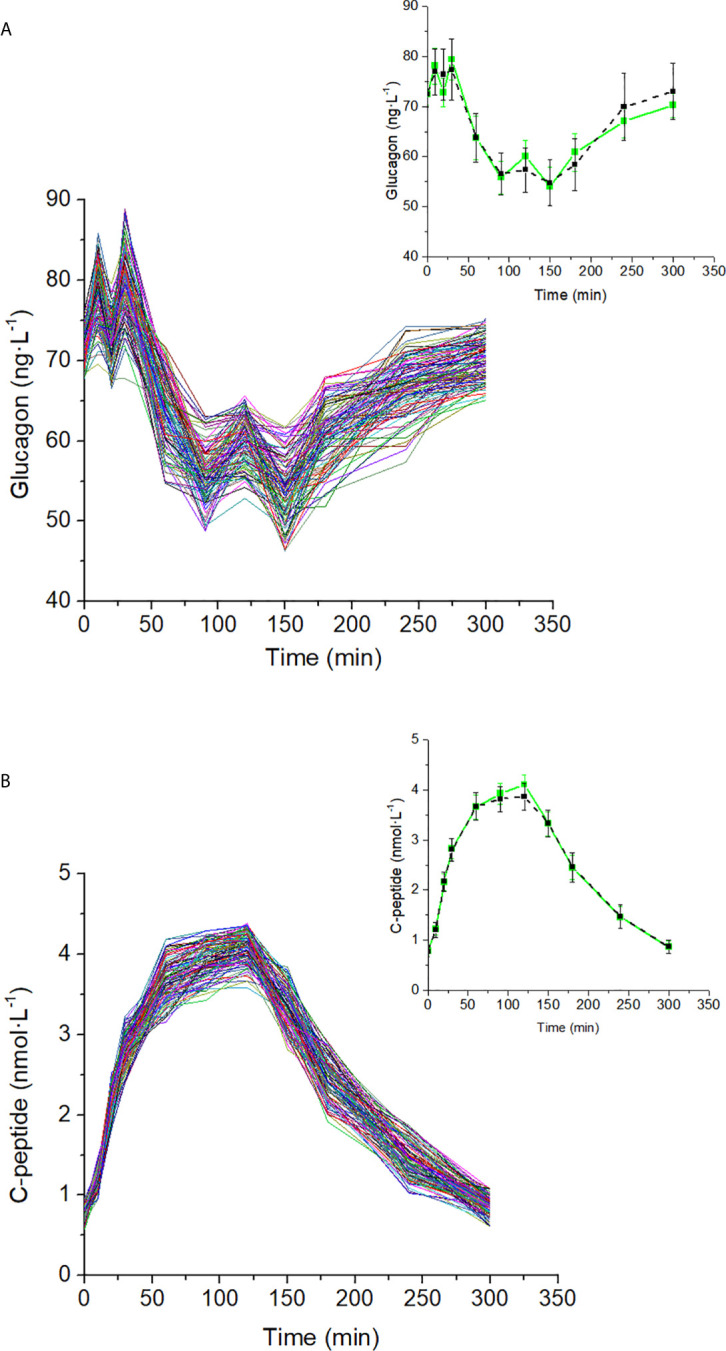
Glucagon **(A)** and C-peptide **(B)** curves in the 100 virtual subjects (spaghetti plot). In the related inset plots, green continuous lines represent the mean (± SD) over the 100 curves; black dashed lines represent mean (± SD) taken from Pepino et al. (31), from which the virtual subjects were derived.

**Figure 5:**
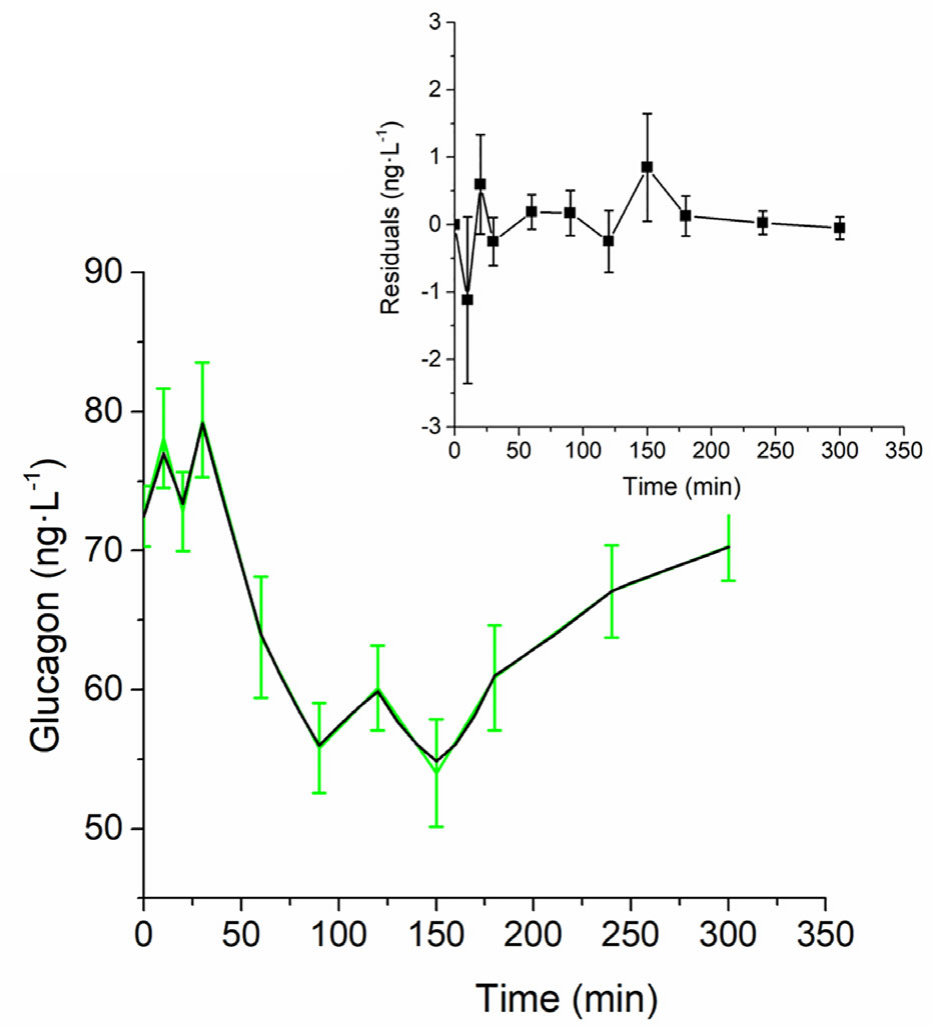
Best-fit results for model validation on the 100 virtual subjects. Green continuous line represents the mean (± SD) over the 100 generated curves; black continuous line represents the mean predicted glucagon curve. Mean residuals over the 100 curves are displayed in the inset plot.

**Figure 6:**
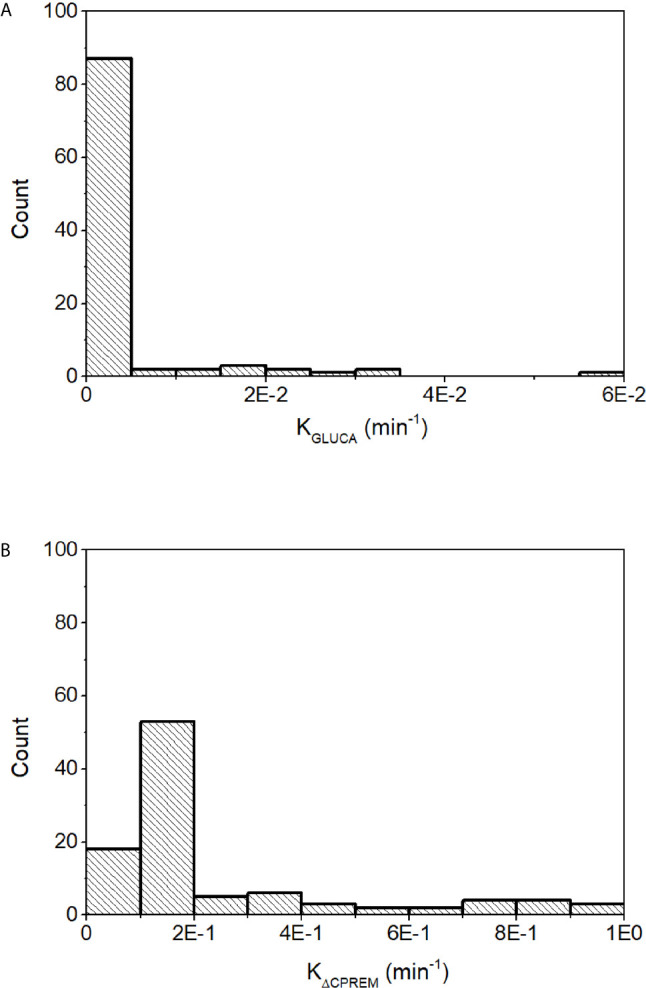
Distribution of K_GLUCA_ **(A)** and K_ΔCPREM_ **(B)** over the 100 virtual subjects.

**Figure 7:**
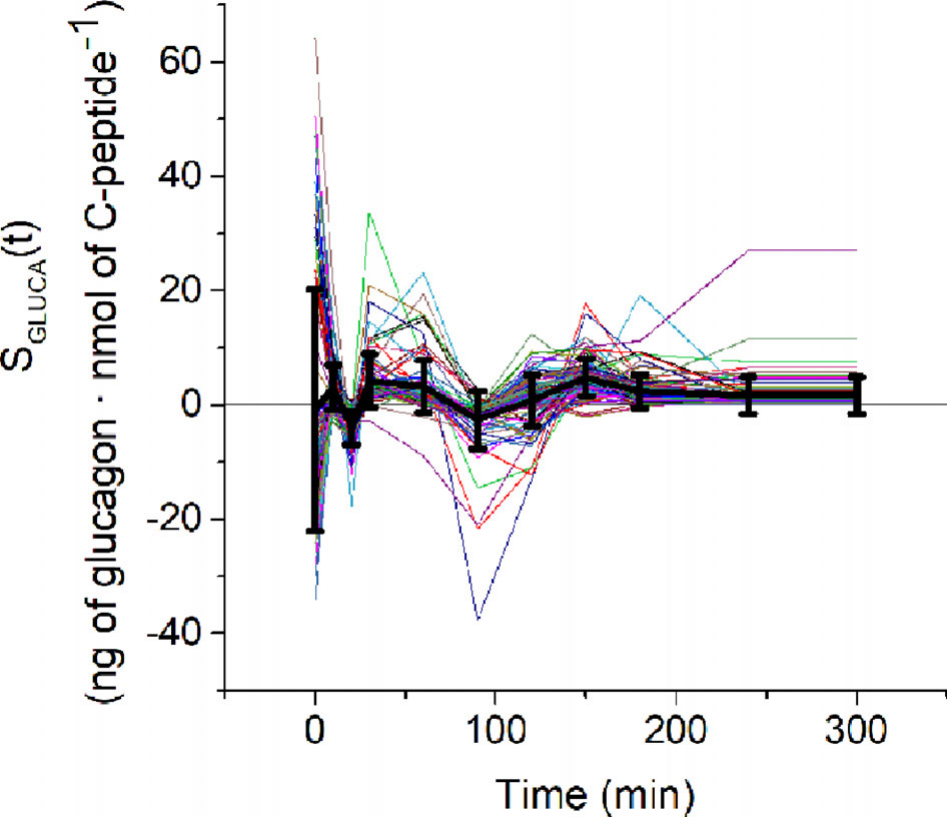
S_GLUCA_(t) estimates over the 100 virtual subjects (spaghetti plot). Black continuous line represents the mean ( ± SD) S_GLUCA_(t).

**Figure 8:**
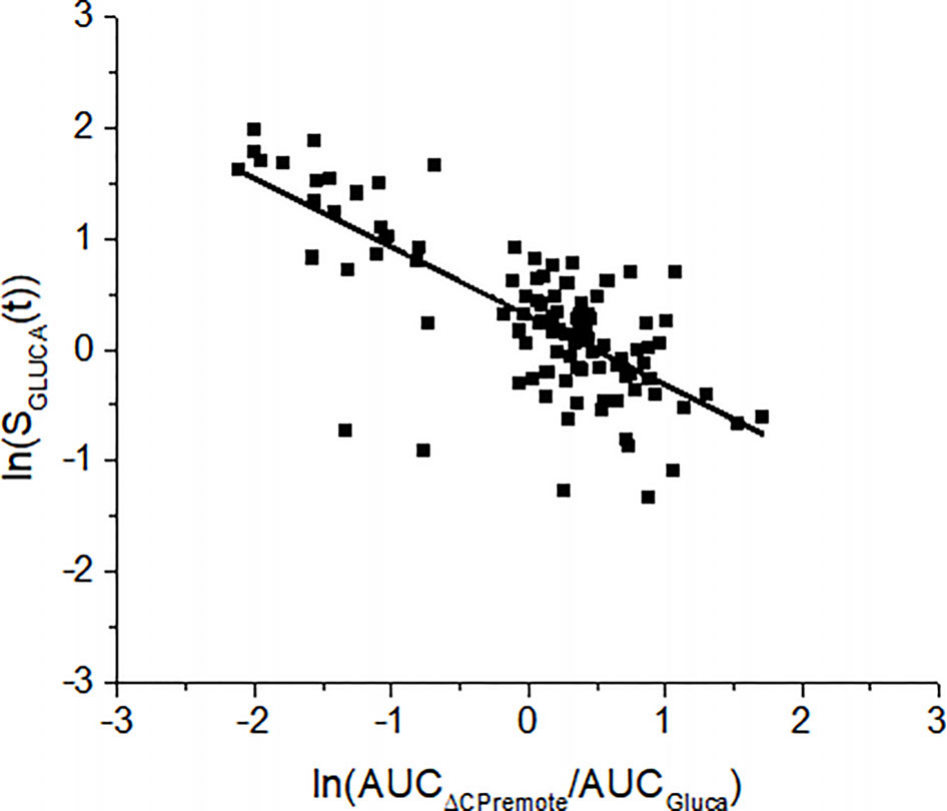
Linear regression analysis between S_GLUCA_(t) and the ratio between the areas under the curve of the remote C-peptide and glucagon (AUC_ΔCPremote_/AUC_Gluca_) over the 100 virtual subjects. Analysis has been performed on the log-transformed values. Regression line equation is: y= -0.6215·x+0.2987. This means that a change of 1 unit in log transformed AUC_ΔCPremote_/AUC_Gluca_ causes a change of log transformed S_GLUCA_(t) by 0.6215.

Glucagon and C-peptide curves in the 100 virtual subjects are shown in **Figure 4**. Model validation on the virtual subjects provided the mean best fit and the related residuals shown in **Figure 5**. Distribution of values for K_GLUCA_ and K_ΔCPREM_ over the virtual subjects is shown in **Figure 6**, whereas the S_GLUCA_(t) patterns are reported in **Figure 7**. A negative significant linear correlation (r = -0.74, p < 0.0001, 95% CI: -0.82 – -0.64) has been found between the log-transformed values of S_GLUCA_(t) and AUC_ΔCPremote_ to AUC_Gluca_ ratio over the 100 virtual subjects. Regression plot is reported in **Figure 8**. Regression line slope and intercept was -0.6215 (95% CI: -0.7346 – -0.5084) and 0.2987 (95% CI: 0.2017–0.3956), respectively.

No significant difference has been found between average S_GLUCA_(t) over the full 5-h OGTT and the 3-h OGTT (p = 0.08); in contrast, average S_GLUCA_(t) over the 2-h OGTT has been found significantly different compared to that over the full 5-h OGTT (p < 0.0001).

**Figure 9:**
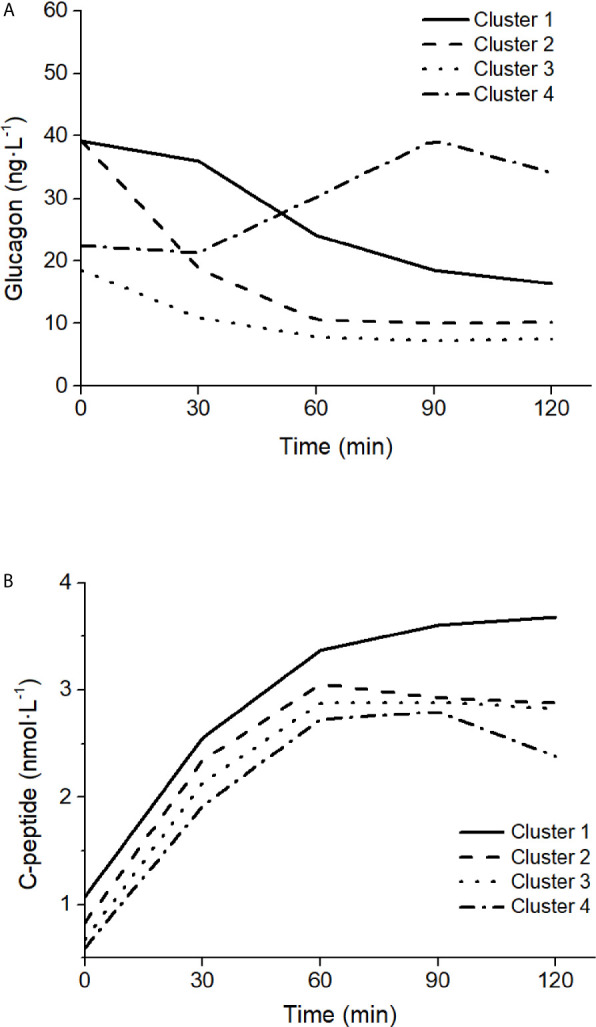
Glucagon **(A)** and C-peptide **(B)** curves in the four clusters of patterns in glucagon kinetics, modified from Gar et al. (38).

**Figure 10:**
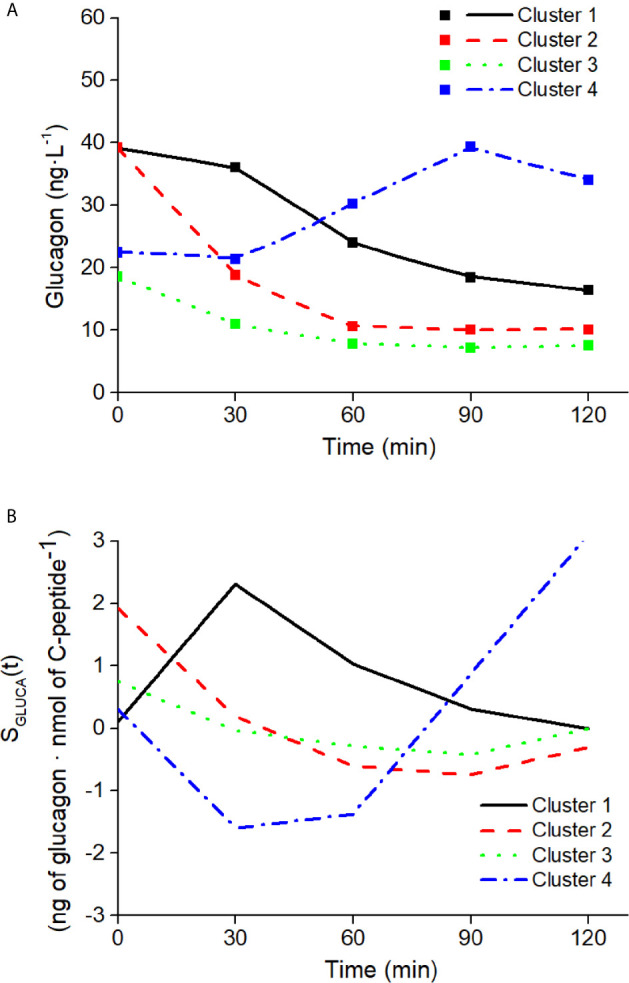
Best-fit results **(A)** and S_GLUCA_(t) estimates **(B)** on the four clusters of patterns in glucagon kinetics. Squares in panel **(A)** are the reference experimental values; lines are the model predictions.

Mean glucagon and C-peptide concentrations for the four clusters, used to assess the ability of the model to capture different patterns in glucagon kinetics, are shown in **Figure 9**. Best-fit results and S_GLUCA_(t) patterns for the four clusters are reported in **Figure 10**.

## Discussion

In this study, we developed a mathematical model of glucagon kinetics during an OGTT, which is a test widely used in the clinical practice for its simplicity, compared to other metabolic tests. The specific characteristics of the model were including parameters with clear physiological meaning and that can be estimated in a single individual, the latter being a crucial feature for potential applications in the clinical context.

Our mathematical model is based on the hypothesis that inhibition of glucagon secretion during an OGTT is mainly determined by glucose-induced insulin secretion due to an intra-islet interaction (5, 6). This led to a simplified description of glucagon regulation, thus disregarding other important regulation mechanisms, one of which is a possible direct effect of glucose (i.e., not only mediated by insulin) (39). Moreover, recent evidence supports the concept of the liver–alpha-cell axis, in which hepatic amino acid metabolism and glucagon secretion are linked in a feedback cycle (40, 41). There is also evidence for insulin secretion being regulated by glucagon action *via* glucagon-like peptide 1 (GLP-1) and glucagon receptors on beta cells (42, 43). Possible regulators of insulin secretion, such as GLP-1, have been considered in other models [also proposed by us (44, 45)] but in the present model we considered insulin secretion as an input signal, regardless of how it has been generated. This simplification, both in the input and in the description of feedback mechanisms, was necessary to achieve our aim to propose a “minimal model”, allowing estimation of glucagon-related parameters in single individuals.

In the study of glucose metabolism, established methodologies exist for the assessment of metabolic aspects of major relevance, such as insulin sensitivity, insulin secretion and possible incretin-based enhancement, and insulin clearance, as assessed for instance in some of our previous OGTT-based studies (45–47). The model of glucagon kinetics presented in this study will have the potential to complement the information derived from an OGTT, provided from the indicated established methodologies. Such new model will add information related to the role of glucagon in maintaining the glucose homeostasis, thus yielding to a more complete picture of the glucometabolic condition of the subjects under study. To our knowledge, this is the first study describing a mathematical model of glucagon kinetics during an OGTT.

Our model of glucagon kinetics simply requires the measure of plasma glucagon and C-peptide. It is based on two ordinary differential equations, and it includes two parameters with specific physiological meaning: the glucagon clearance from plasma, K_GLUCA_, mainly due to liver and kidneys (32, 33), and the sensitivity of the glucagon secretion from the pancreatic alpha cells to the inhibitory effect of insulin, S_GLUCA_(t). In short, this can be named as sensitivity to glucose-induced insulin secretion of the glucagon inhibition and can be denoted as alpha-cell insulin sensitivity. This may parallel the concepts of sensitivities of the beta cells, such as the established concept of beta-cell glucose sensitivity (48), and the more recently proposed beta-cell incretin sensitivity (49).

In addition to S_GLUCA_(t), the model also includes one further parameter, i.e., the clearance of C-peptide from a compartment remote with respect to plasma. It should be acknowledged that the physiological interpretation of this parameter may not be possible, since such remote compartment cannot be defined precisely. In fact, the concept of a compartment remote from plasma is not new in mathematical models of glucose metabolism, being used as an example in the well-known original Minimal Model (for the assessment of insulin sensitivity and glucose effectiveness from an IVGTT) (50), and more recently in other models, such as our model for the assessment of non-esterified fatty acids kinetics (51). However, though it is sometimes believed that the compartment remote from plasma may be identified in the interstitial fluid, this may not be totally correct. Indeed, the remote compartment should be seen as a mathematical trick, without strict physiological meaning, which is useful to introduce a time delay between the action of the input/forcing variable and the effect on the output variable (i.e., C-peptide and glucagon, respectively, in the presented model), for a better description of the system under analysis. Therefore, K_ΔCPREM_ has precise physiological meaning (clearance), but it is applied to a variable (remote C-peptide) that is not clearly physiologically defined.

In our approach, we hypothesized that the glucagon inhibition during the OGTT is due to the action of the secreted insulin, as suggested in several studies and summarized in some reviews, such as (4). However, in the model we exploited plasma C-peptide, rather than insulin. This is due to the reason that C-peptide may be more accurate than insulin for the assessment of insulin secretion, since they are secreted equimolarly, but the former does not significantly undergo partial degradation from the liver. Thus, we hypothesized that plasma C-peptide may be more adequate than insulin as marker of insulin secretion, i.e., to assess its inhibitory effect on glucagon. This appeared confirmed by the data of the study (31). Indeed, in linear regression analysis over the average data of the analyzed population in study (31), we verified that C-peptide time samples were significantly inversely related to those of glucagon, whereas those of insulin (as well as those of glucose) were not. In addition, surprisingly, insulin secretion values, as assessed in study (31) were similarly not related to glucagon. This may be due to limitations in the method used for insulin secretion assessment, and/or to the fact that we analyzed average curves, rather than individual curves that were not available. Moreover, it should be acknowledged that insulin may inhibit glucagon secretion by stimulation of somatostatin secretion rather than through a direct effect on the alpha cells (52). Nonetheless, above all, the reported findings suggest that the choice of plasma C-peptide as marker of the insulin effect on glucagon may be the most reasonable option: on one hand, not requiring further mathematical modeling for the calculation (as it is for insulin secretion assessment), and on the other hand showing more strict relationship with glucagon compared to both plasma insulin and insulin secretion.

In our model, sensitivity to insulin of glucagon inhibition was defined as a time-variant parameter, S_GLUCA_(t), differently to glucagon and remote C-peptide clearance, K_GLUCA_ and K_ΔCPREM_, respectively, which were assumed constant during the OGTT. This choice was based on the consideration that estimating an average clearance of glucagon (and of remote C-peptide) during the OGTT is sufficient for our purposes, whereas at contrast the sensitivity to insulin of glucagon inhibition has to be assessed with higher accuracy, being the parameter of major interest in our approach. Thus, S_GLUCA_ was defined as varying at each time sample of the OGTT reported in the study (31). This choice may arise questions about the identifiability of our model parameters, which is a crucial issue for the possibility to estimate the parameters in single individuals and hence for the potential clinical applicability of the model. To this purpose, we first performed *a priori* identifiability analysis. We found that, if the model assumes constant S_GLUCA_, absolute *a priori* identifiability is obtained. If the model assumes the time variant S_GLUCA_, absolute identifiability is lost, but still local identifiability is reached, meaning that there is a finite number of solutions of the minimization problem for the estimation of the parameters. Moreover, to further reducing the uncertainty in parameters estimation, we exploited the concept of regularization, that is, the process of adding information for the solution of a possibly ill-posed problem thus preventing overfitting (53), similarly to what was done in previous studies (45, 48). Indeed, in the cost function minimized by the nonlinear least-squares solver we included further factors, in addition to the traditional sum of squares of the difference between data and model prediction (fit). First, we assumed that S_GLUCA_(t) cannot undergo excessively rapid variations, as this would be unphysiological: thus, in the cost function we added a term to penalize the entity of S_GLUCA_(t) second-order derivatives. In addition, we assumed that S_GLUCA_(t) should typically show positive values, though some negative values are sometimes possible: thus, we included a term penalizing the number of S_GLUCA_(t) negative values. Furthermore, among the regularization factors we included K_GLUCA_. This means having assumed that during the OGTT the clearance of glucagon is small, based on the reasonable hypothesis that, during the test, the sensitivity to insulin of glucagon inhibition has more influence than glucagon clearance for glucagon disappearance. Of note, such constraint explains the small values observed for K_GLUCA_. The described regularization strategy allowed us to overcome the problem of incomplete *a priori* identifiability, and to include physiologically-based constraints for greater reliability and improved meaning of the estimated model parameters [especially with regard to S_GLUCA_(t)].

Despite negative values of S_GLUCA_(t) are penalized, they are allowed in our model approach, this meaning that glucagon inhibition by insulin may not be effective in some time periods. This appears in fact clearly indicated by the inspection of average curves of study (31). Indeed, such curves suggest that during an OGTT glucagon may increase in some time periods, though slightly, whereas C-peptide (as well as insulin) is not decreasing as one would expect, thus indicating that in those periods the relationship between insulin action and glucagon variations is lost. A clear explanation for this phenomenon is still elusive, but non-suppressed (increasing glucagon) during OGTT may surprisingly be associated to even healthier metabolic phenotype (less hepatic fat, higher insulin sensitivity) according to some studies, such as (54).This may be also due to reason that, despite insulin is often reported as the major determinant of glucagon inhibition (4), other studies suggest a possible direct effect of glucose (i.e., not only mediated by insulin) (39), as well as several other factors (4, 39). It should also be observed that in different glucose tolerance tests, such as a mixed meal test, the effect of such factors other than insulin may be even more relevant. From this point of view our model, accounting for possibly negative values of the sensitivity to insulin of glucagon inhibition, appears adequate for future model developments, also including other factors that may influence glucagon inhibition (though adding further variables may affect the clinical applicability, and hence should be considered with caution).

Another aspect that we addressed in the model development is the choice of the initial condition of the parameters to be estimated. To this purpose, we exploited the MultiStart algorithm (36). We are aware that other approaches may be possible such as genetic algorithm strategy, as done in some of our previous studies (55–57). In this study, we opted for the indicated approach, as it appeared somehow simpler to implement. Similar problem was the choice of the optimal weights of the regularization factors. However, since for some technical difficulties it appeared hard to exploit the MultiStart algorithm for such aspect as well, in this case we simply randomly generated different values for the weights.

In our study, we also computed the 95% CI of the estimated parameters [though for brevity we presented results only for the case of the data derived from the study (31)]. We found that CI of K_ΔCPREM_ did not include the zero value, probably due to its small estimated values, whereas K_GLUCA_ did. As regards S_GLUCA_(t), during the OGTT the algorithm estimated six positive values and four negative values. For the positive values, the CI did not include zero, thus indicating the robustness of the positive value estimations. For the negative values, two did not include zero as well, whereas the other two included, this indicating that for those two values the estimation was somehow uncertain. On the other hand, it should be acknowledged that these findings are related to average data, rather than actual individual human data, and this may have an effect on CI calculation. In addition, as the used method for CI calculation required the exploitation of the Jacobian matrix provided by the solver, it cannot be excluded that the tolerance in the matrix accuracy numeric (rather than analytic) calculation may have an effect as well.

Our model proved to perform properly, as indicated by the good fit for each virtual subject (considering the tolerance that we assumed in relation to the accuracy and precision of plasma glucagon measurement), and by the physiologically plausible values of the estimated parameters, varying within a range appearing reasonable, especially with reference to S_GLUCA_(t). In addition, it should be noted that, as expected, we found significant relationship between mean S_GLUCA_(t) and mean C-peptide to glucagon ratio, this further indicating reliability and robustness of our model approach. Our model approach can also be easily extended to the study of subjects characterized by different metabolic phenotypes. In fact, the model was successfully tested on characteristic OGTT curves as representative of different clusters of glucagon kinetics (low or high fasting glucagon; rapid or delayed suppression).

We also found interesting results regarding the model performances in relation to different OGTT durations. Indeed, data used in our study are related to a 5h-OGTT (31), which is not usually performed in clinical settings. However, when testing sensitivity of the S_GLUCA_(t) estimation to OGTT duration, in the population of virtual subjects our approach provided comparable results with data limited to 3h-OGTT; in contrast, it appears that the present approach cannot be extended to shorter OGTT (2h). Analysis over 2 h limits glucagon kinetics only to the suppression phase, thus neglecting the phase in which glucagon restores to the basal conditions, thus it is not surprising that 2h-OGTT did not provide results comparable to those of the 5-h OGTT. Nonetheless, since our model is not constrained to work with a specific OGTT duration, we tested it on five time-point 2h OGTT curves, which have been identified by Gar et al. as representative of different clusters of glucagon kinetics (38). As shown, our model was able to reproduce all characteristic patterns observed during the five time-point OGTT. Thus, if we aim to consider the complete glucagon kinetics we have to resort to 3h-OGTT, but if we are interested only to the suppression phase we can limit analysis to 2h-OGTT, and to five time-point only (*i.e.*, the typical 2h-OGTT time samples: 0, 30, 60, 90, 120 min).

Comparison of our findings to those of previous studies is difficult. As previously outlined, some mathematical models were developed with the aim to investigate glucagon secretion at cellular level (12–21), whereas other models, more similarly to ours, were developed for whole-body analyses, but mainly for simulation purposes rather than for clinical applications (22–25). Other studies presented models of glucagon kinetics for possible clinical applications, but they were focused on the analysis of glucagon administered exogenously, or for the analysis of the not common glucagon challenge test (26–29). The study having more aspects in common with ours is that of Kelly et al. (30). In that study, a comprehensive model of glucagon kinetics and dynamics was developed for possible clinical applications, i.e., for possible assessment of model parameters in individuals. That model has merits in the details of the physiological phenomena analyzed. For instance, the model allows to address aspects not analyzed in our study, such as the ability of glucagon to promote hepatic glucose production, sometimes denoted in study (30) as glucagon sensitivity. However, that model, which is to some extent an extension of the traditional Minimal Model (50), applies to glucose tolerance tests other than the OGTT, such as the IVGTT (consistently with the field of application of the Minimal Model). In addition, both the model versions (based on nonlinear or linear relationships between glucose, glucagon and insulin) are relatively complex and including several parameters (five equations with 11 unknown parameters, and four equations with 10 unknown parameters, for nonlinear and linear model versions, respectively). Thus, doubts may arise whether all these parameters are identifiable in a single individual. Of note, analyses of model identifiability were not presented. As regards the concept of sensitivity to insulin of glucagon inhibition, which is the focus of our study, it has to be acknowledged that the study (30) addresses the issue as well, though it does not appear the aspect of major interest in the presented analyses. In fact, one parameter similar our S_GLUCA_(t) is presented, defined as the maximum rate at which insulin suppresses glucagon secretion. However, in study (30) the parameter is assumed constant, thus not considering its possible variations during a test. In addition, and most importantly, study (30) presents simulations where such parameter was varied in a given interval, whereas the estimation of the parameter based on glucagon and insulin data is not reported, and hence it was not proved that the model may allow estimation of such parameter. Thus, to our knowledge our study is the first showing the possibility to assess the sensitivity to insulin of glucagon inhibition in single individuals, having proved the feasibility of S_GLUCA_(t) estimation with individual data curves.

In conclusion, we developed a model of glucagon kinetics during the OGTT, with special interest for the sensitivity to insulin of glucagon inhibition, denoted as alpha-cell insulin sensitivity. Strength of our model is simplicity and the possibility to estimate parameters with clear physiological meaning (i.e., the parameters of glucagon kinetics) in a single individual, thus being potentially adequate for use in clinical settings. Future investigations may consider the option to introduce some model improvements, including description of further factors possibly affecting glucagon suppression during the OGTT, but paying attention to avoid model approaches not adequate for possible clinical applications. Another aspect for future studies will be the assessment of the actual clinical relevance of our model approach, by studying populations including subjects with different degree of glucometabolic impairment.

## Data Availability Statement

The datasets for this study include already published data and virtually generated data. The virtually generated data are available upon request to the corresponding author.

## Author Contributions

MM and AT conceived and designed the study. MM, and AT analyzed and interpreted the data. LB, CG, BA, and GP validated the analysis. MM and AT wrote the first draft of the manuscript. LB, CG, and BA critically revised the manuscript. All authors contributed to the article and approved the submitted version.

## Conflict of Interest

The authors declare that the research was conducted in the absence of any commercial or financial relationships that could be construed as a potential conflict of interest.

## Abbreviations

OGTT, oral glucose tolerance test; T2DM, type 2 diabetes; IVGTT, intravenous glucose tolerance test; SD, standard deviation; CI, confidence interval.
