# Supplementary figures and images for: Mathematical Model of Glucagon Kinetics for the Assessment of Insulin-Mediated Glucagon Inhibition During an Oral Glucose Tolerance Test
Source: Front Endocrinol (Lausanne). 2021 Mar 22;12:611147. doi: 10.3389/fendo.2021.611147 (PMC8020816; doi:10.3389/fendo.2021.611147)

# Supplementary Material

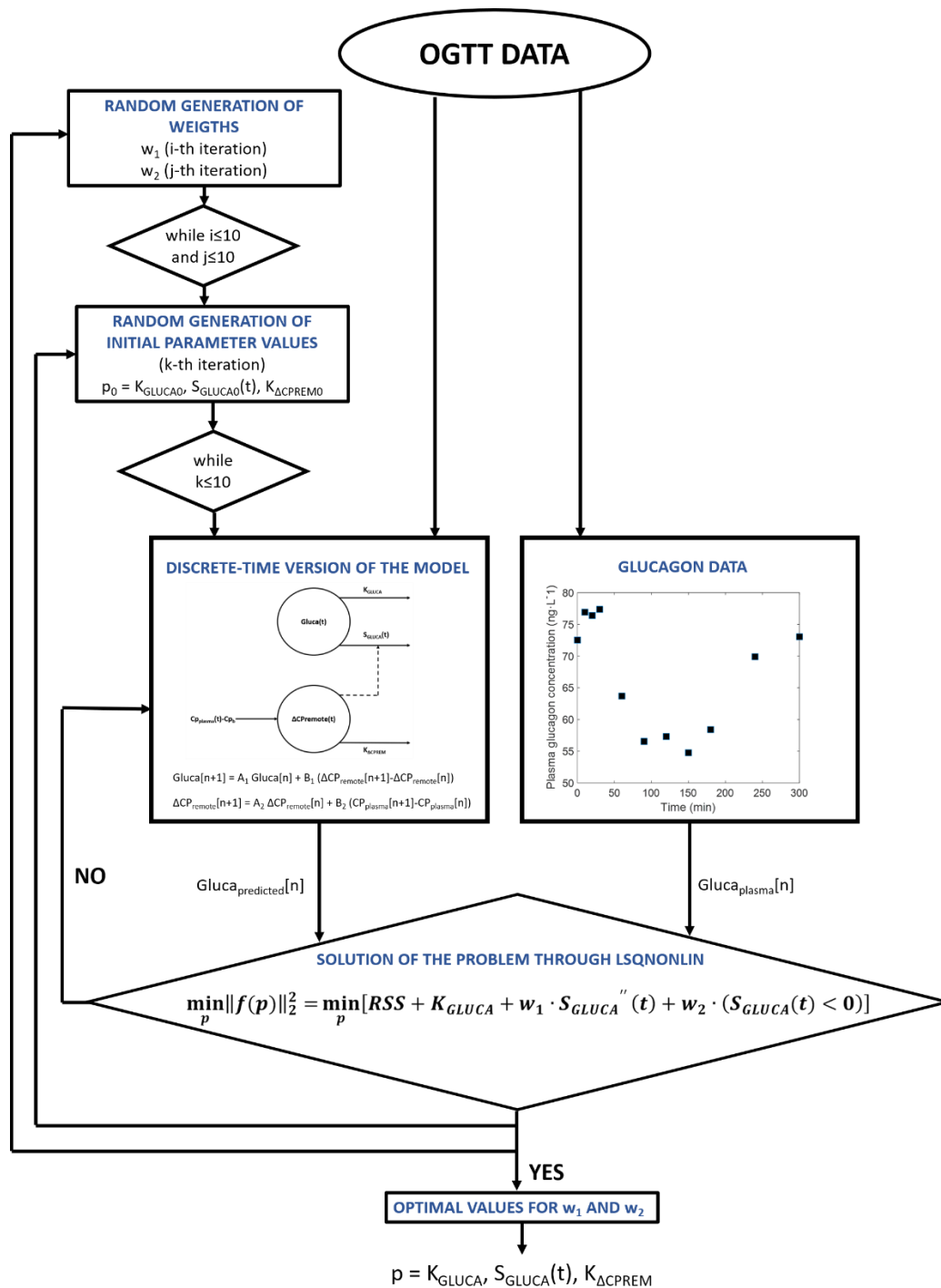

Supplementary Figure 1. Model implementation.

Supplement: Supplementary file 1 [file Image_1.pdf]
